# Effects and implementation of a minimized physical restraint program for older adults in nursing homes: A pilot study

**DOI:** 10.3389/fpubh.2022.959016

**Published:** 2022-09-06

**Authors:** Jun Wang, Weichu Liu, Xuelian Li, Luyong Li, Jinyan Tong, Qinghua Zhao, Mingzhao Xiao

**Affiliations:** ^1^Department of Urology, The First Affiliated Hospital of Chongqing Medical University, Chongqing, China; ^2^Department of Gynecology, The First Affiliated Hospital of Chongqing Medical University, Chongqing, China; ^3^Chongqing Shanxing Nursing Home, Chongqing, China; ^4^Department of Nursing, The First Affiliated Hospital of Chongqing Medical University, Chongqing, China

**Keywords:** physical restraint, nursing homes, nursing staff, complex intervention, educational program

## Abstract

**Purpose:**

Physical restraint (PR) reduction interventions are currently exploring in developed regions with well-established welfare systems, whereas developing countries with fast population aging have not attracted enough attention. This China's pilot study evaluated the effects of a minimized PR program on restraint reduction and nursing assistants' knowledge, attitudes, intention, and practice toward PR and explored nursing assistants' experience of the program.

**Patients and methods:**

This was a one-group, pretest, and posttest pilot trial with a nested qualitative descriptive study. A minimized PR program was obtained by summarizing the best evidence and was implemented in one Chinese nursing home with 102 older adults from December 18, 2020, to March 21, 2021. An educational program including three theoretical lectures and one operation training was first conducted for nursing assistants one-month period. The primary outcome was PR rate at 3 months. The secondary outcomes contained duration of restraints, types of restraints, the rate of correct PR use, the incidence of falls and/or fall-related injuries, and antipsychotics use at 3 months. Data on PR use and older adults' characteristics were collected through physical restraints observation forms and older adults' medical records. Nursing assistants' knowledge, attitude, intention, and practice toward PR were measured using the Staff Knowledge, Attitudes, and Practices Questionnaire regarding PR at 1 month. A semi-structured interview for two administrative staff and a focus group discussion with 13 nursing assistants were analyzed using content analysis to explore perspectives of intervention implementation at 3 months.

**Results:**

There were a significant increase in knowledge, attitude, and practice and a decrease in intention of nursing assistants after 1-month educational intervention (*P* < 0.001). Furthermore, only the rate of correct PR increased and the duration of restraint in the daytime decreased significantly at 3 months (*P* < 0.05). There were no significant effects on PR rate and other secondary outcomes at follow-up. Qualitatively, nursing assistants demonstrated overtly supportive perspectives and that assistance from the program enhanced their knowledge and practice. They noted several challenges that impeded implementation.

**Conclusion:**

The intervention has acknowledged some benefits and was valued by nursing assistants. Implementation barriers should be addressed before delivering in larger trials.

## Introduction

According to an international consensus statement, physical restraint (PR) was defined as “any action or procedure that prevents a person's free body movement to a position of choice and/or normal access to their body by using any method that is attached or adjacent to a person's body and that they cannot control or remove easily” (1). PR is commonly applied in the clinical environment despite numerous evidence presenting its lack of efficacy and safety and even cause of accident events and/or injuries (2–4). Compared with other populations, older adults are more vulnerable to PR and PR-related adverse consequences (e.g., the decline in ability, depression, social isolation, and loss of self-esteem or sense of worth) (5–9), and the prevalence of PR for older adults in long-term care facilities has been reported to be 84.9% (10). Thus, reducing PR and alleviating poor outcomes constitute a critical component of care quality to improve the wellbeing of older adults.

Our previous bibliometric analysis (11) showed that over one-third of research on PR was corresponding to strategies for reducing the use of PR, and most of them focused on countries with better welfare systems, such as Germany, Norway, and the Netherlands. Currently, PR management comprises mental and behavioral management projects (12), quality improvement projects (13), restraint alternatives (14), educational programs (15), and multi-component interventions (16, 17). The last two of these strategies are the most common interventions. The educational programs were administered over a period of 1–6 months, including educational sessions ranging from one to ten sessions with a total of 6–10 h (18, 19). These differences in education period, times, delivery, and content may affect the effectiveness of educational programs. Huizing et al. (20) found that PR training for nursing staff was not significant in reducing the use of PR. Nevertheless, several studies have revealed that PR training could change the attitudes and practice of nursing staff and reduce the utilization of restraint among older adults (21, 22). Previous systematic reviews (2012 and 2017) have evaluated the effects of educational intervention on reducing PR, but it has not been concluded (18, 23). A recent systematic review emerged on the effectiveness of the educational interventions at the endpoint study in reducing or preventing the use of PR in nursing homes (19). These findings support the implementation of educational programs. Sure enough, the educational program is the foundation and only a starting point; other interventions are needed. Thus, multi-component interventions that include PR education are evolving.

With the increasing research on multi-component interventions containing education, the effectiveness of these complex interventions in reducing the use of PR has attracted much attention. In 2012, Köpke et al. (16) developed guideline-based multi-component interventions and found that the PR rate in the intervention group was lower than that in the control group, decreasing from a baseline of 31.5–22.6%. Their team optimized the previous interventions and conducted a pragmatic cluster randomized controlled trial in 120 nursing homes. There is no significant difference in the effects of reducing PR between the two multi-component intervention groups, both of which reduced the utilization rate of PR and had no significant impact on improving the quality of life of older adults (17). Education is a core part of these multi-component interventions and also incorporated consultation, institutional policies, alternatives, etc. The implementation of multi-component interventions required many resources. Thus, the cost of multi-component interventions has become an obstacle to wider dissemination, particularly in developing countries with imperfect welfare systems. However, whether these interventions are feasible targets for countries with serious aging but imperfect welfare system as effective interventions for PR reduction is rarely assessed.

To our knowledge, previous studies on reducing PR among older adults have been conducted in Hong Kong and Taiwan regions, with few studies in China mainland (24–26). Nevertheless, these studies have described single interventions such as training and alternatives with a small sample size study design and reached inconclusive evidence. Due to these deficiencies, it remains unclear whether educational or multi-component interventions are effective and feasible for older adults in Chinese nursing homes. Previously, we have identified the incidence of and risk factors for PR (27), nursing staff's knowledge, attitudes, and practice (KAP) (28). Findings revealed the lack of practical norms of restraints in long-term care and staff's knowledge deficits and training needs. Additionally, existing norms are less instructive for staff in long-term care facilities. For instance, a group standard named *Nursing Practice Standards Physical Restraint in Inpatients* issued by the Chinese Nursing Association is applied to registered nurses in various hospitals and other medical institutions that can refer to the implementation (29). PR use is only restricted by the National Mental Health Law when applied to patients with mental disorders in China's mainland (30). Thus, it is necessary to develop reasonable minimized PR programs for older adults in long-term care, especially based on the best evidence summary.

Although several previous studies have developed interventions based on comprehensive surveys, as well as made process and outcome evaluations (17, 31), the practice of PR varies across survey contexts and cultural backgrounds, such as social values, nursing environment, and care modes of older adults. Therefore, it is necessary to develop suitable interventions for Chinese older adults in long-term care. This pilot study was to inform future multicenter large-scale randomized controlled trials that implement a minimized PR program, through the following aims:

(a) Describe the potential effects of interventions on the use of PR for older adults (i.e., PR rate, duration of PR, the rate of correct PR use, types of PR, and the occurrence of adverse events).
(b) Identify the impacts of training on a minimized PR program on nursing assistants' KAP toward PR use.
(c) Explore nursing assistants' experiences and perceptions of intervention implementation processes after program completion.

## Patients and methods

### Design, setting, and participants

This was a one-group, pretest, and posttest pilot trial with a nested qualitative descriptive study. A qualitative descriptive study was performed to describe the nursing assistants' perspectives of the program (32). A three-month intervention was carried out from December 18, 2020, to March 21, 2021. The study was prospectively registered in the Chinese Clinical Trial Registry (ChiCTR2000040741). The study protocol was approved by the Ethics Committee of the First Affiliated Hospital of Chongqing Medical University (No. 2019-107) and complied with the Declaration of Helsinki.

This pilot study was conducted in one private nursing home in Chongqing, China. This nursing home consisted of a geriatric ward and an orthopedic rehabilitation unit. This minimized PR program was applied to the geriatric ward. One hundred and fifteen beds were located in this area, mainly for the disabled, semi-disabled, and demented older adults. The healthcare team comprised two doctors, four nurses, one rehabilitation therapist, and 24 nursing assistants. There were no principles and standards guiding staff to use PR on older adults in the nursing home, and staff did not receive formal training on PR previously. All older adults who were present in the nursing home during the study period were included, as well as newly admitted older adults during follow-up who underwent the practice of intervention, for the program was a cluster trial in the nursing home. The exclusion criteria were older adults (a) who were absent in the nursing home on the day of data collection, (b) who were told to be not allowed to observe because of serious and special illnesses, and (c) with involuntary movement. A single-group rate difference test prior to power analysis in the G^*^ Power 3.1 software was performed to calculate the sample size (33). The required sample size was 87 based on an alpha error of 0.05 and a power of 0.80. By consulting similar studies and baseline investigation of this nursing home (16, 27), the endpoint rate was assumed as 0.23 and the baseline rate was 0.37. Thus, samples of older adults in the geriatric ward met the requirements.

### Minimized PR program development

**Figure 1:**
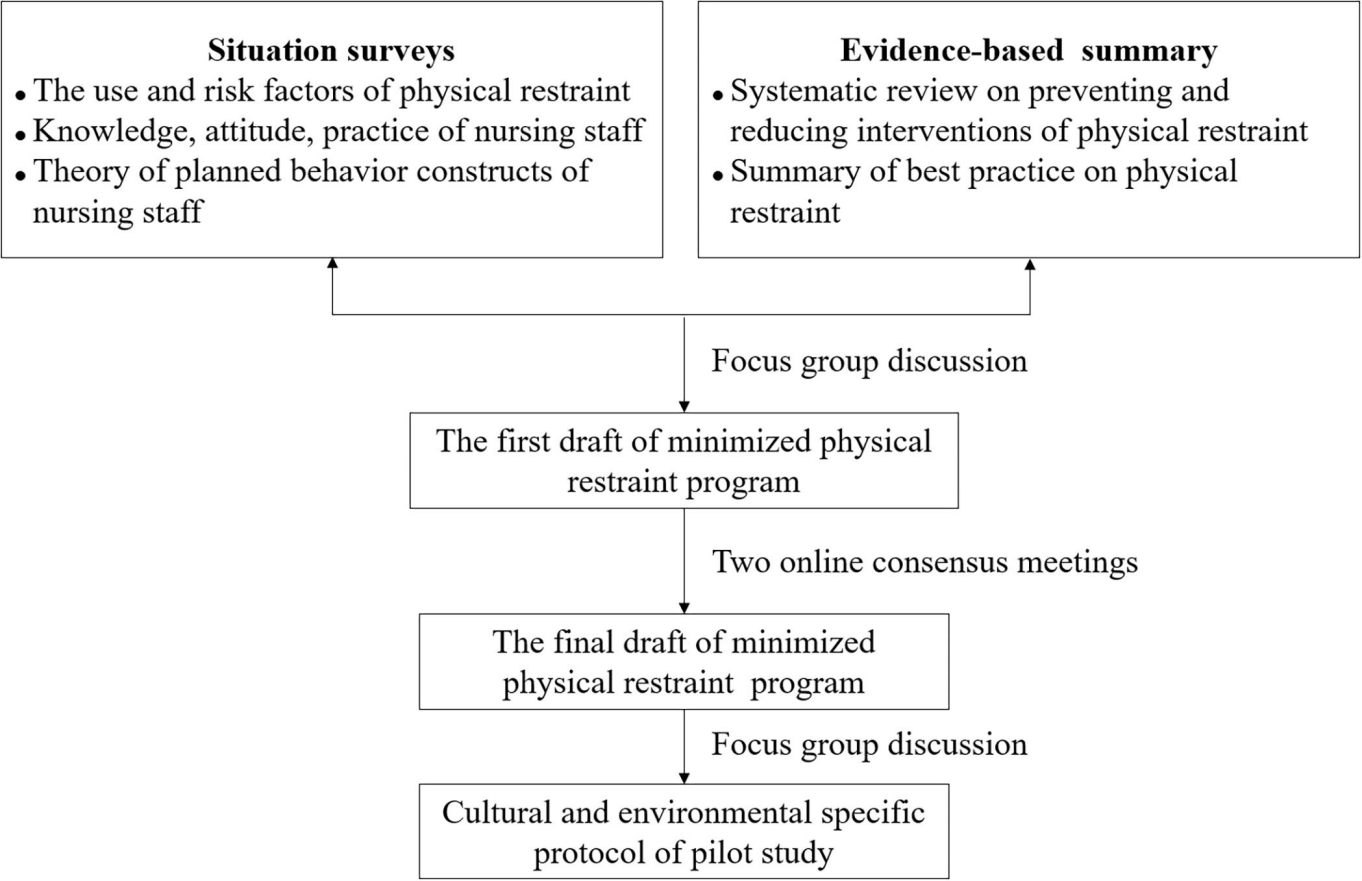
Minimized PR program development flowchart.

The minimized PR program of this study was developed in three steps (Figure 1). First, the study was started by summarizing the best evidence for PR use in nursing homes. A total of 17 pieces of literature were finally included, and details are provided in Supplementary Appendices 1, 2. Surveys were also conducted during this phase to determine the prevalence of and risk factors for PR among older adults in nursing homes, as well as the knowledge, attitudes, intention, and practice of nursing staff. The aim was to gain an in-depth understanding of the risk factors for PR and nursing staff's knowledge deficits and training needs in the current situation. Then, the first draft of the program was formed based on concretizing and complementing the best evidence combined with clinical practice issues and background. Next, two online consensus meetings with 10 experts in the fields of geriatric care, PR, quality control, and rehabilitation medicine were held to revise the program. Ultimately, the program included 29 items, divided into 13 dimensions, comprising use principles, organizational support, personnel requirement, evaluation, decision making, informed consent, alternatives, implementation, monitoring, removing, recording and reporting, and personnel training. Details of the minimized PR program are provided in Supplementary Appendices 3, 4.

### Intervention process

Supplementary Appendix 5 presents the components of the intervention. A previous study reported that knowledge deficits and evidence-based education insufficiency were the main barriers to reducing PR use in this study's location (28). Thus, an educational program regarding the content of the minimized PR program was conducted for nursing assistants one-month period before the implementation of the program. Table 1 presents the components of the educational program. Nursing assistants who directly took care of older adults and implemented PR attended the training. The four nurses in the nursing home stayed in the ward to look after older adults during training periods and did not participate in the training. Four training sessions were arranged over 1 month, including three theoretical lectures and one operation training, and performed by the team's nurse specialist. To ensure nursing assistants understand the training content, we complied with the following principles: (a) making simple slides with cartoons or photographs and performing trial lecture in advance, (b) performing straightforward Chongqing dialect, interactive question discussion, and real examples sharing, and (c) collecting suggestions to improve in the next session. In addition, during the training period and the subsequent implementation period of the minimized PR program, the research team offered PR practice consultation for 2 h per week to the nursing assistants in the nursing home. The investigator and the head nurse supervised the implementation of the plan throughout the entire process. The researcher team visited the nursing home one time or two times a week during the first and second months of the intervention and held a symposium in the second month of the intervention to solve problems in the intervention process. In the third month, due to strict isolation management of COVID-19, the researchers could not visit on-site and communicated with the nursing manager online to supervise, feedback, and solve problems. During the study period, the problems that nursing assistants asked for help with were as follows: (a) alternatives recommendation for PR, (b) neuropsychiatric symptoms management in dementia, (c) evaluation of the necessity to use PR, (d) handling of two older adults who fell, and (e) stress relief of individuals with fear of responsibility.

**Table 1:** Education elements for nursing assistants in medical–nursing facilities.

Education period	Every Tuesday at 14:30, 4 consecutive weeks, 40–60 min each time.
Education content	**1. Week 1: Overview of physical restraint** The definition of physical restraint, the use and influencing factors, the adverse consequences, and the concept of minimized physical restraint, etc. **2. Week 2: Nursing practice for physical restraint** Principles, process, assessment, decision making, informed consent, implementation, monitoring, recording, and restraint release. **3. Week 3: Physical restraint alternatives** The concept of alternative measures, specific alternative methods in terms of physiology, psychosocial, nursing management, environment, etc., and the effect of alternative measures. **4. Week 4: Skills practice toward physical restraint** A video about how to use restraint for patients, 10 min; operation demonstration of limb restraint and waist restraint, 15 minutes; nursing assistants practice and evaluation, 30 min.
Education materials	Make slides for three theoretical lectures in the form of photographs, literature data, and real cases. Practical operation video sourced from “***Patient Restraint Methods***” section of “***50 Common Nursing Operation Techniques***” published by the Chinese Medical Association.
Education recipient	All nursing assistants in the nursing home fully participated in education.

### Outcomes and data collection

The primary outcome of this study was to assess the PR rate among older adults in the facility. The secondary outcome contained (a) duration of restraints, types of restraints, the rate of correct PR use, the incidence of falls and/or fall-related injuries, and the use of antipsychotics at the older adults level and (b) knowledge, attitudes, intention, and practice toward PR, experience, and perspectives of intervention implementation processes at the level of nursing assistants.

The PR rate was defined as the ratio of the number of older adults who were restrained at least once on the day of data collection to the total number of older adults. The use of PR was collected by direct observation, which was reported to be the most reliable method for collecting data on PR use (34). Two trained observers collected the data at four points, that is, in the morning (10:00), noon (13:00), afternoon (16:00), and evening (20:00). The observation time was determined because older adults were most likely to be active at times from 9:30 to 11:30 or 15:00 to 17:00, rest from 12:30 to 14:30, and go to bed at nearly 19:00. During the 1 week before and after the intervention, four visiting dates and time point for each day were determined randomly and not communicated to the facility staff.

Another three restraint-related outcomes were measured by observers. First, the duration of restraint in 24 h was the sum of restrained time of four observation intervals. Standardized interviews with the direct nursing assistants of older adults were adopted, and the nursing assistants were asked for how long older adults were restrained during the interval between each observation. Second, all types of restraint used with older adults were recorded by observers. Third, the rate of correct PR use indicated the ratio of older adults who were restrained correctly to all restrained older adults. Situations of improper use of PR were as follows: (a) not meeting the restraint indications, (b) being too loose, (c) being too tight, (d) violating the functional position of older adults, (e) fixing on the bed rail, (f) observing restraint-related complications, and (g) other occasions of improper use. Additionally, data on falls and fall-related injuries and use of antipsychotics for 3 months before intervention and a 3-month intervention period were collected from medical health records. The above outcomes related to older adults were measured at the pre- and post-intervention time. All these data were collected using a customized data collection form.

The Chinese version of the Staff Knowledge, Attitudes, and Practices Questionnaire regarding PR was performed to assess KAP toward PR of nursing assistants. The questionnaire was developed by Janelli et al. (35) and validated in Hong Kong nursing homes by Suen (36). The intention subscale of the PR Theory of Planned Behavior (PR-TPB) questionnaire was used to evaluate the intention toward PR of nursing assistants. These two instruments were validated in this study's location, and more details could be found in the previously published papers (37). The Cronbach's alpha coefficients of knowledge, attitude, intention, and practice subscale were 0.756, 0.689, 0.638, and 0.800, respectively. The questionnaire reported appropriate reliability according to the suggested level of a Cronbach's alpha coefficient ≥0.60 of subscales (38). This information was collected before the first education class and at the end of the last education class.

Data regarding the older adults' characteristics including gender, age, length of residence, number of chronic diseases, cognitive function, degree of care dependency, mobility, fall risk, and tube indwelling tube were obtained from the older adults' health records and their ability assessment data. Cognitive function was derived from the Mini-Mental Status Examination (MMSE) (39). The degree of care dependency was determined by the Barthel index (40). Mobility was measured by the items “activity” and “mobility” of the Braden scale (41), a widely used instrument for the assessment of pressure ulcer risks. The Morse scale was used to assess the older adults' fall risk (42). Details were presented in our previous publication (27).

Experience and perspectives of intervention implementation processes for nursing assistants were obtained from a qualitative descriptive study conducted after the 3-month intervention. The qualitative study was comprised of (a) focus group interviews with 13 nursing assistants who participated in PR training and the implementation of the minimized PR program and (b) a semi-structured interview with two administrators of a nursing home (i.e., one dean of nursing home and one nursing manager of the geriatric ward). Of 24 nursing assistants in the nursing home who attended the training, five nursing assistants always cared for restraint-free older adults and did not directly practice the program, four were not willing to participate in interviews, and two requested leave to return home during data collection. Thus, a total of 13 nursing assistants attended the focus group interviews. Focus groups are often used to develop research hypotheses or interventions, find out problems encountered in research practice, etc. This method can collect a large amount of interactive data in a short period of time, making up for the insufficiency of traditional questionnaires. In addition, two administrators provided organizational support and leadership during the practice of the program. Therefore, through semi-structured interviews with these two administrators, we can understand the impact of the program from the perspective of managers. Due to no beginning conceptual or theoretical framework to guide and focus the initial interview questions, organizing scheme of concepts developed from the literature and group discussion helped in guiding our data collection (32). Major areas of interviews included (a) positive and negative experiences in the process of program implementation, (b) the overall evaluation of the program, such as convenience, acceptance, and feasibility, and (c) recommendations for larger implementation of the program. The interview guide is presented in Supplementary Appendix 6. The interview was presided over by a researcher who had experience in the qualitative study. Interviews were conducted in a comfortable and quiet room of the nursing home on the third day after the intervention finished and audio-recorded.

### Data analyses

Data were analyzed using SPSS software version 25.0. The Shapiro–Wilk test was performed to identify normality for continuous variables. A descriptive analysis of the variables was performed using percentages for categorical variables, mean (***M***) and standard deviation (***SD***) for continuous variables with a normal distribution, and median and interquartile range (***IQR***) for skewed data. The knowledge, attitude, intention, and practice scores of nursing assistants conformed to the normal distribution, and the paired ***t***-test was used for comparison. The duration of restraint was examined using the Wilcoxon signed-rank test because of the non-normality of the data. The count data were compared using the chi-square test or Fisher's exact test. ***P*** < 0.05 was set for statistical significance. As data on the semi-structured interview were obtained from only two managers, it is difficult to extract the themes independently with limited information. Considering that the focus group and semi-structured interview involved similar interview questions, we integrated data for analysis. The audio-recorded interviews were transcribed verbatim within 48 h, and the transcription was checked against the audio records by two researchers to ensure their accuracy. Content analysis was performed to analyze the words and phrases of the interview data (43). Data analysis started with reading all data repeatedly to achieve immersion and obtain a sense of the whole. Then, read word by word to code by highlighting the words that captured key opinions. Next, make notes and categorize codes based on links and differences between codes. Transcription of record and text encoding of the qualitative research data was performed in NVivo 12.0 software.

## Results

### Quantitative findings

#### Participant characteristics

**Figure 2:**
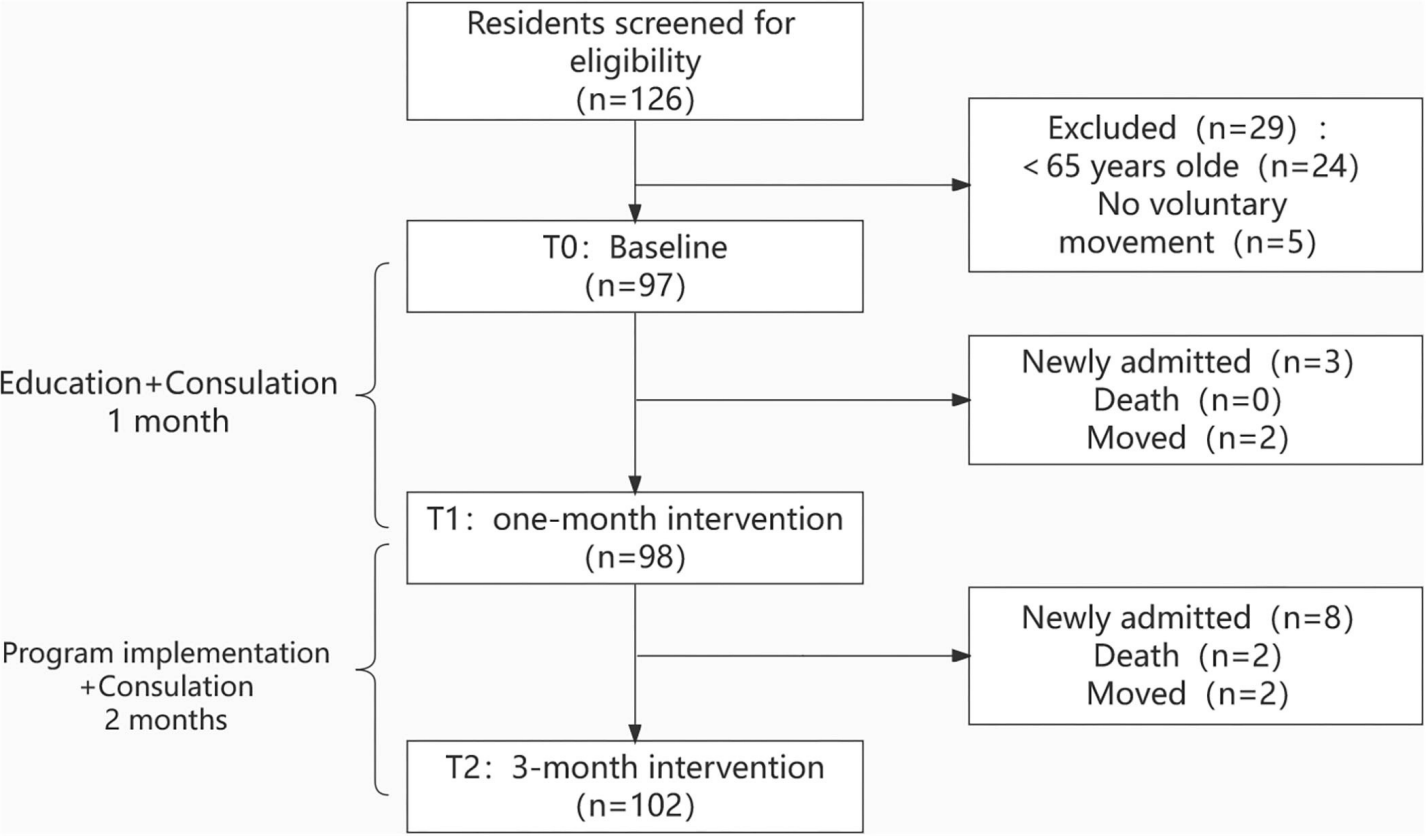
Participants' flowchart.

Of 126 older adults who were screened, 29 were excluded based on the eligibility criteria, and a flowchart of included participants is displayed in Figure 2. In the intervention period, 11 older adults were newly admitted, two died, and four left the nursing home. Thus, a total of 102 older adults were included after the 3-month intervention. Table 2 presents the older adults' characteristics. Older adults' age ranged from 66 to 100 years, with a mean age of 84.10 years (SD = 5.86). Fifty-six (54.90%) older adults were female, and 24 (23.53%) were indwelling tubes. Fifty-nine (57.84%) older adults suffered moderate-to-severe cognitive impairment.

**Table 2:** Baseline information of older adults in the study (***n*** = 102).

**Variables**	***n***	**Percentage (%)**
**Gender**
Male	46	45.10
Female	56	54.90
**Age (years)**		0.00
≤ 70	16	15.69
71–80	28	27.45
81–90	48	47.06
≥91	10	9.80
**Length of residence (years)**
≤ 1	17	16.67
1.1–3	48	47.06
3.1–5	32	31.37
>5	5	4.90
**Mobility (points)**
8	6	5.88
7	9	8.82
6	18	17.65
5	34	33.33
4	20	19.61
<4	15	14.71
**Indwelling tube**
Yes	24	23.53
No	78	76.47
**Number of chronic diseases**
<3	24	23.53
3–5	61	59.80
≥5	17	16.67
**Care dependency (points)**
61–100	15	14.71
41–60	40	39.22
≤ 40	47	46.08
**Cognitive impairment**
Intact	15	14.71
Mild	28	27.45
Moderate	34	33.33
Severe	25	24.51
**Physical agitation**
Yes	13	12.75
No	89	87.25
**Verbal agitation**
Yes	18	17.65
No	84	82.35
**Fall risk**
Low risk	18	17.65
Moderate risk	45	44.12
High risk	39	38.24

A total of 24 nursing assistants including eight males and 16 females were engaged in the educational program, aged from 48 to 62 years, with a mean age of 54.58 years (*SD* = 3.56). Ten nursing assistants received middle school education, and 14 were educated in primary school and below. The working years in the nursing home ranged from 1 to 6 years, with an average of 4.00 (*SD* = 1.41). They were responsible for 5.29 (*SD* = 1.04) older adults and paid 3,000 to 4,000 RMB per month.

#### Outcomes of older adults

**Figure 3:**
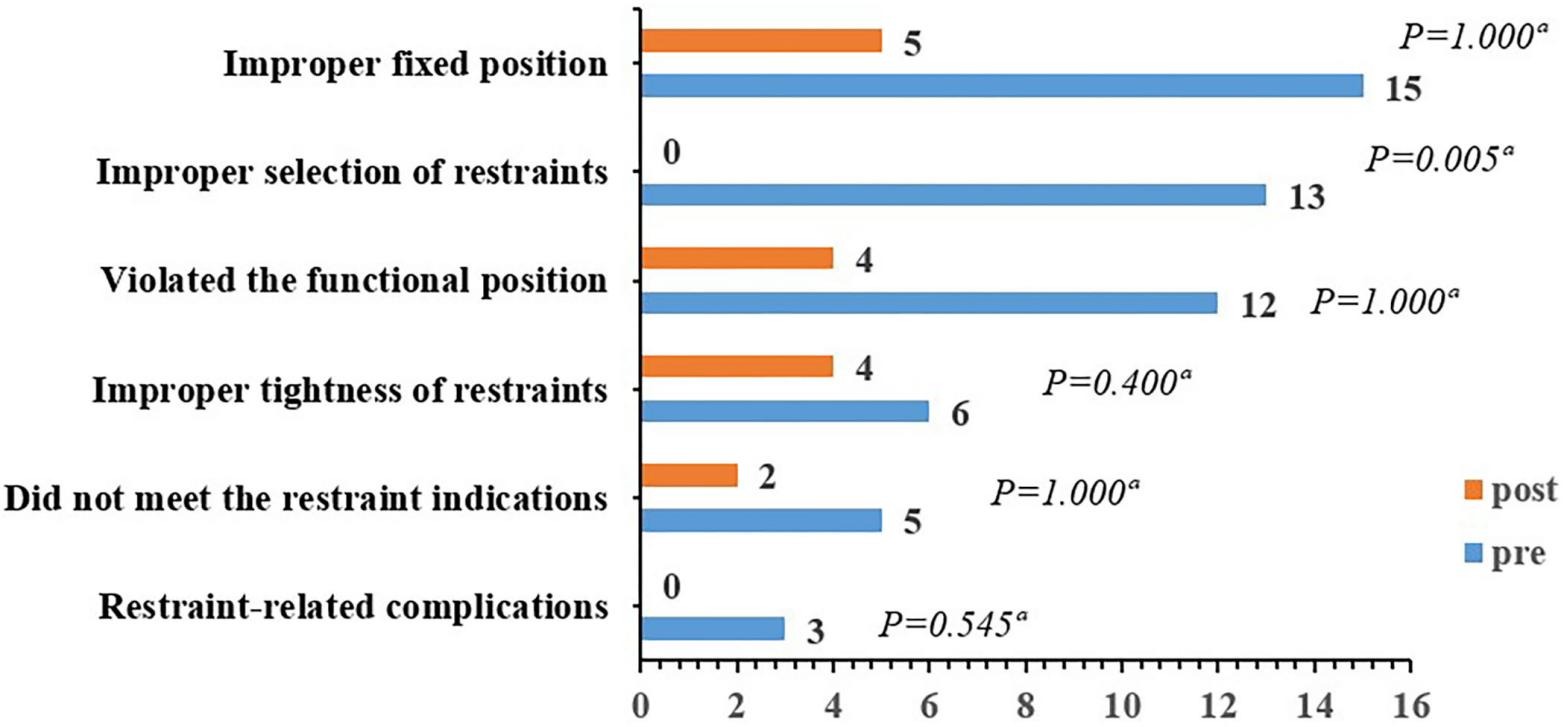
Comparison of baseline and 3-month type of incorrect restraint use for restrained older adults. ^a^Fisher's exact test.

**Figure 4:**
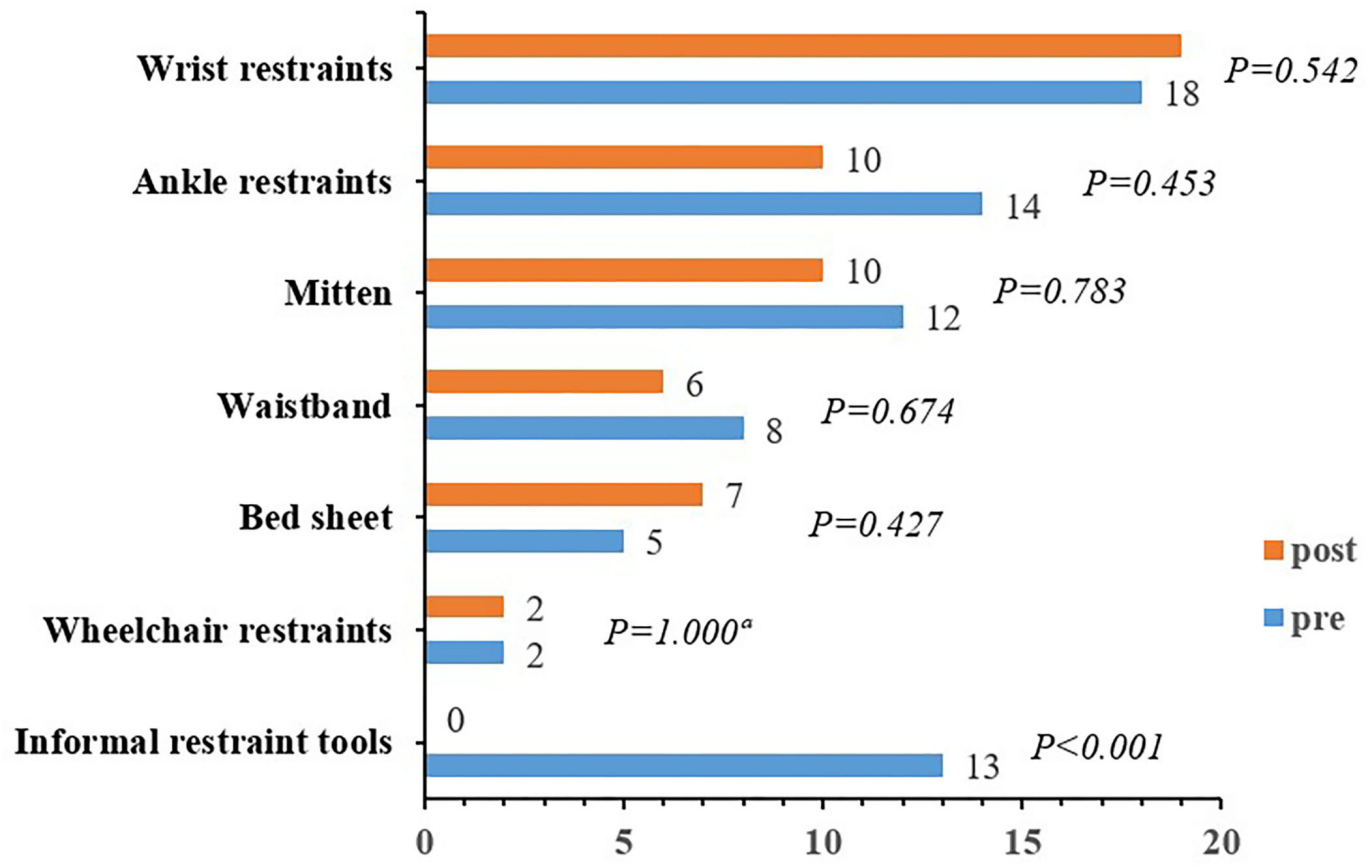
Comparison of baseline and 3-month restraint type for restrained older adults. ^a^Fisher's exact test. Informal restraint tools include hemp rope, work gloves, socks, etc.

A comparison of baseline and 3-month outcomes for older adults is displayed in Table 3. The primary outcome (PR rate in the nursing home) was 38.14% (37/97) at baseline and 33.33% (34/102) after the 3-month intervention. No significant difference in PR prevalence was found pre- and post-intervention (*P* = 0.479). The duration of restraint in 24 h, daytime, and nighttime of older adults was not following the normal distribution, and the Wilcoxon signed-rank test was used for pre- and post-intervention comparison. The duration of restraint in the daytime decreased rank significantly from baseline to follow-up (*P* = 0.030), whereas the duration of restraint in 24 h and nighttime had no significance (*P* > 0.05). The rate of correct PR use of 73.53% (25/34) after the 3-month intervention was significantly higher than that at baseline with a correct rate of 35.14% (13/37). The number of older adults with at least one fall or fall-related injury, as well as antipsychotics use, did not differ statistically significantly before and after the intervention (*P* > 0.05). A comparison of baseline and 3-month types of incorrect restraint use for restrained older adults is illustrated in Figure 3. Except for the improper selection of restraint tools with statistically significant result (*P* = 0.005), no significant difference in other types of incorrect restraint use was displayed before and after the intervention (*P* > 0.05). Thirteen older adults were restrained using informal restraint tools at baseline, such as hemp rope, work gloves, and socks, which were eliminated after the 3-month intervention (*P* < 0.001) (Figure 4).

**Table 3:** Comparison of baseline and 3-month outcome measures for older adults.

**Variables**	**Pre-intervention**	**3-month intervention**	**Statistic**	***P*-value**
Physical restraint (*n*)	37	34	0.501	0.479
Duration of restraint in the 24 h^a^	15 (13, 18)	14 (13, 16)	−1.468	0.142
Duration of restraint in the daytime^a^	6 (4, 8)	4.5 (3.75, 6)	−2.165	0.030
Duration of restraint in the nighttime^a^	10 (8.5, 11)	10 (8, 11)	−0.563	0.574
Rate of incorrect use^b^	13/37	25/34	10.500	0.001
Fall/fall-related injury (*n*)	3	2	0.003^c^	0.955
Use of antipsychotics (*n*)	17	19	0.041	0.840

a Median (interquartile range) for description, Wilcoxon's signed-rank test for comparison.

b Restrained correctly/restrained older adults.

c Continuous calibration of chi-square.

#### Outcomes of nursing assistants

The knowledge, attitude, intention, and practice toward PR of nursing assistants conformed to the normal distribution. The paired *t*-test findings (Table 4) showed that the scores of nursing assistants' PR knowledge, attitude, and practice after the 3-month intervention increased significantly compared with baseline (*P* < 0.001). Additionally, the total score of intention toward PR decreased significantly compared with baseline, indicating nursing assistants less intend to use PR on older adults (*P* < 0.001).

**Table 4:** Comparison of baseline and 1-month physical restraint knowledge, attitude, intention, and practice of nursing assistants (*n* = 24).

**Variables**	**T0, Mean (SD)**	**T1, Mean (SD)**	**T1-T0, Mean (SD)**	***t***	***P***
Knowledge	4.00 (1.22)	7.46 (1.06)	3.46 (1.64)	10.323	<0.001
Attitude	28.21 (1.56)	33.75 (1.62)	5.54 (1.98)	13.727	<0.001
Intention	17.54 (1.35)	13.50 (1.14)	−4.04 (1.65)	23.407	<0.001
Practice	24.25 (1.70)	34.79 (1.84)	10.54 (2.21)	−11.966	<0.001

T0, pre-intervention; T1, after 1-month intervention; SD, standard deviation.

### Qualitative findings

#### Participant characteristics

A total of 13 nursing assistants including six males and seven females participated in the focus group interview, all aged from 48 to 62 years, with a mean age of 52.92 years (*SD* = 4.18). Five nursing assistants received an education level of primary school and below, and eight educated in middle school. They worked in the nursing home for over 3 years. A semi-structured interview was conducted with two managers. One was the dean of the nursing home, male, aged 53 years, and educated for undergraduate, and one was the nursing manager, female, aged 32 years, educated for undergraduate, and working for 8 years.

#### Experience and perspectives of intervention implementation processes

Three themes, namely, learning and applying for the program, experiencing two-side feelings, and encountering challenges and barriers, were identified, including seven sub-themes as follows: knowledge update, attitudes changes, practice improvement, confidence in the program, burden increases, difficulty to assess the necessity of restraint, and poor feasibility of alternatives. The complete list and reflective quotes are given in Supplementary Appendix 6.

## Discussion

To our knowledge, this is the first study to develop and assess the effectiveness and feasibility of the minimized PR program based on a comprehensive current investigation and the best evidence summary of Chinese older adults in nursing homes. This pilot and feasibility study may support a potential value in a multi-component intervention among older adults receiving long-term care services even though the prevalence of PR in the nursing home was not significantly decreased. On the whole, the intervention was supportive by the nursing assistants, and the improvement in the staff's KAP was significant. However, some barriers to implementation should be addressed before conducting large trials or routine clinical practice.

### Effects of the program on older adults

Several remarkable results (16, 17, 44) have been obtained in RCTs conducted abroad with multi-component interventions; however, inconsistent finding was observed in this study. In line with these studies, the minimized PR program implemented in this study was a set of multi-component interventions, including organizational support, education support, and consultation. The difference is that this study covers comprehensive nursing practice in the whole implementation process, and the previous programs emphasize the prevention of risk factors, with a less specific implementation process involved. The reasons for the lack of effectiveness in the primary outcome (i.e., PR rate) should be addressed. First, the pilot study was conducted in one nursing home with small sample size, and most of the older adults were severely disabled and limited in mobility and cognitive impairment. It might be more difficult to completely remove restraints in a population with a high risk, especially in a short time. Second, direct observation of data collection may result in measurement bias, and omission of the restrained older adults may affect the result, especially in small sample size. These may contribute to no significant change in PR rate before and after the intervention. Furthermore, PR training for nursing assistants was performed only four times, and for a short time, two previous studies conducted different educational components targeting all relevant persons. Although the training content in this study was comprehensive and systematic, the effectiveness of the education was affected by complex factors that may affect routine clinical practice, which has been confirmed in many studies (20, 23). Evidence-based practice (EBP) requires continuous improvement and is affected by various factors, such as leadership, insufficient time, and resources (45). The deep-rooted notion that PR is regarded as a protective measure for preventing injuries and accidents needs to experience the long road to change. In this study, there is no significant change in PR rate after 3 months of evidence-based practice, suggesting that more efforts and measures may be needed to drive practice change. In future, models and theories of implementation science are recommended to plan, guide, and assess the implementation of the minimized PR program (46).

The minimized PR program in this study improved the rate of correct use of PR, which was consistent with the results of evidence-based practice of PR in ICU patients in China (47). Before the intervention, the improper use of PR was mainly due to obvious violation of functional position and improper fixed position, such as lower limbs of older adults hanging in the air when the ankle restraint was used, as well as the restraint belt and gloves, was fixed on movable objects (e.g., bed rails). The implementation dimension in the program specified the measures of fixation, tightness, body position, and other measures, so these problems have been significantly improved after the application of the program. Regarding the choice of restraint tools, 13 older adults used informal restraint tools such as socks and work gloves to restrain their fingers and hemp ropes to bind limbs or waist, which may lead to great potential safety hazards. As expected, the use of informal restraint tools decreased to zero after program implementation. Compared with this study, education combined with consultation alone in the previous study did not change the types of restraints (48). It suggests the value of the program to the reasonable and standardized use of PR.

Several studies have shown that prolonged PR may further lead to a decline in ability and cognitive function among older adults (3, 49). This study found a reduction in the duration of restraint in the daytime after the intervention, which shows a positive effect of the program. No significant change in nighttime restraint duration was observed, which may be related to the limited nursing energy of nighttime care and the continuous restraint of older adults for fear of accidents. Most older adults in the nursing home suffered cognitive impairment, often accompanied by nocturnal behavior and other mental and behavioral symptoms, which increases the possibility of restraint in the nighttime. Additionally, insufficient nighttime staff is another potential reason. Consistent with previous studies, adverse outcomes (i.e., falls and fall-related injuries and antipsychotic use) had no significant increase before and after the intervention (16–18).

### Effects of the program on nursing assistants

The quantitative research results of this study found that training significantly improved the PR knowledge, attitudes, and practice of nursing assistants in the nursing home and weakened the intention to use PR. A lower intention score indicates a lower willingness to use PR for older adults. This is in line with the findings of a study of hospital nurses in Malaysia (50). In accordance with previous studies (50–52), educational interventions improve staff's PR knowledge, attitudes, and practice. Nevertheless, Huizing et al. (53) reported that staff's PR knowledge and practice in the experimental group improved significantly after intervention and no significance was observed compared with the control group. Previously, staff in the nursing home did not receive PR-related training and had little understanding of restraint practice. The qualitative results revealed that the educational intervention deepened the nursing assistants' theoretical understanding and improved their cognition and practical ability toward PR to a certain extent. The latest evidence-based training with restraint-free culture goes beyond other approaches that were restricted to fragmentary knowledge (24, 25). Formal and continuous training with evidence-updated content is suggested for in-service education in the nursing home.

### Feasibility and challenges to the program

From the aspect of older adults, the minimized PR program improves the correct rate of use, reduces the duration of restraint, and avoids adverse consequences caused by prolonged PR or improper restraint. It reveals that the program is in line with the rights and interests of older adults and is acceptable for more wide nursing homes. In the sight of nursing assistants and managers, qualitative interviews showed that the program provided a reference for the PR practice of nursing assistants, and evidence-based practice regulated the use of restraint, which promoted the improvement in nursing quality in the nursing home. Our previous study reported relatively high prevalence and poor routine practice, and it is an urge to guide the clinical practice based on this updated program (27). Therefore, other nursing homes could formulate action strategies according to the program and carry out practical changes combining the actual culture and working environment.

Some challenges should be addressed, and further improvement is needed. Negative experiences existed in the process of implementation of the program, mainly concerning unexpected risks and increased caregiving workload. On the one hand, it suggests that we need to further improve the measures, formulate action strategies, strengthen the safety protection of older adults without using restraints, and reduce the psychological pressure on nursing assistants. On the other hand, the transformation of evidence into clinical practice requires changing the original working mode and process, which requires more time and energy for the nursing assistants. Lack of time, knowledge, and skills is the main obstacle to evidence-based practice (31, 54). It indicates that the opinions of practitioners should be actively listened to in later practice, and the nursing assistants and older adults-centered coping strategies should be formulated to mobilize their enthusiasm and improve their behavioral compliance, thereby promoting the maintenance of practice change (55). Besides, nursing assistants pointed out that the need for restraint was difficult to assess and that alternative measures of restraint were difficult to practice. In clinical practice, there is a lack of PR assessment tools suitable for the environment of the nursing home, and the comprehensive assessment of risk factors by validated tools for specific risk factors to identify older adults at risk of PR was recommended. We are developing machine learning-based models to predict the risk of PR in older adults, which could help medical staff in the early identification and PR management of older adults. Additionally, few studies on the effect and specific implementation of restraint alternatives were conducted. Although we trained nursing assistants for using alternatives, the approaches were not specific and easy to implement, especially for the effectiveness of alternatives. Moreover, the process of alternative implementation was not evaluated in this study. These limitations directly result in the difficulty in evaluation and alternative practice toward PR. In future, it is vital to develop a specific model of care that promotes the best support for the implementation of alternative approaches to the use of PR.

### Strengths and limitations

In developed regions with well-established welfare systems, an evidence-based intervention for preventing and reducing PR use in nursing homes has been systematically explored. There is a paucity of studies examining the effects of interventions on PR reduction and staff's KAP improvement in Chinese nursing homes, especially based on the program developed by summaries of best practices. This study, based on a minimized PR grogram obtained by shreds of evidence, is the first to determine the effects and feasibility of a minimized PR program implementation process. Several limitations should be addressed. First, the methodological limitation is that this study is a one-group, pretest, and posttest pilot trial with a small sample size in one nursing home to test the initial applicability of the program. In the qualitative part, the saturation of the data cannot be assessed due to the limited number of interviews. Data were only collected for 4 days rather than continuous records, which may lead to measurement bias, although direct observation was extensively used in studies. The challenges found in the preliminary application of the program should be solved and contributed to continuous quality improvement, thereby further conducting a large-sample controlled trial with a more rigorous study design. Second, the follow-up time was limited to a 3-month study period, and the long-term effectiveness of the minimized PR program in PR use prevention and reduction is still inconclusive. Third, evaluation and alternatives of PR were challenges and barriers to the program implementation due to the limitations of existing evidence and research, which deserves further study. In addition, a pre-planned family's participation in the minimized PR program was not carried out as the recruitment of older adults' family members was not successful because of strict containment as a result of COVID-19. The minimized PR program was a multi-component, complex intervention, and evidence-based practice of the program requires a certain amount of human, material, and financial resources. In future, we should focus on the most effective ingredients and simplify the program, which will help popularize clinical implementation.

## Conclusion

Few studies have discussed evidence-based measures that minimize PR use among older adults in Chinese nursing homes. Quantitative findings demonstrated that the minimized PR program could increase the correct use of PR and decrease the duration of restraint in the daytime, as well as promote the nursing assistants' knowledge, attitude, and practice toward PR. Qualitatively, the minimized PR program was considered a supportive approach to enhancing staff's perspectives and practice on PR and standard the use of PR among older adults in nursing homes. Through the educational intervention, education and consultation assisted with the decision making and implementation of PR use and bolstered the nursing assistants' confidence to overcome several dilemmas to use PR correctly. Collectively, these findings reveal that staffs in nursing homes are supportive of utilizing evidence-based interventions as an approach to preventing and reducing PR use among older adults. Nevertheless, further research is necessary to test the long-term compliance and sustainability of the program and expand large studies in nursing homes.

## Data availability statement

The data presented in the study are included in the article/Supplementary material, further inquiries can be directed to the corresponding author/s.

## Ethics statement

The studies involving human participants were reviewed and approved by the Ethics Committee of the First Affiliated Hospital of Chongqing Medical University (No. 2019-107). The patients/participants provided their written informed consent to participate in this study.

## Author contributions

JW, MX, and QZ performed substantial contributions to conception and design, interpretation of data, manuscript drafting, and manuscript revising. WL, XL, LL, and JT were involved in data collection and literature review. All authors have read and critically revised the manuscript for its intellectual content and approved the final version.

## Funding

This study was funded by the National Key Research and Development Program of China (2020YFC2005900), Postgraduate Research and Innovation Project of Chongqing Province (CYS19202), and Key Project of Chongqing Science and Technology Commission (cstc2018jscx-mszd0030).

## Conflict of interest

The authors declare that the research was conducted in the absence of any commercial or financial relationships that could be construed as a potential conflict of interest.

## Publisher's note

All claims expressed in this article are solely those of the authors and do not necessarily represent those of their affiliated organizations, or those of the publisher, the editors and the reviewers. Any product that may be evaluated in this article, or claim that may be made by its manufacturer, is not guaranteed or endorsed by the publisher.
