# Supplementary material for: Effects and implementation of a minimized physical restraint program for older adults in nursing homes: A pilot study
Source: Front Public Health. 2022 Sep 6;10:959016. doi: 10.3389/fpubh.2022.959016 (PMC9486015; doi:10.3389/fpubh.2022.959016)
Supplement: Supplementary file 1 [file Data_Sheet_1.docx]

Supplementary Material

**Supplementary appendix 1 A detailed process of the evidence summary**

**Supplementary appendix 2 Overview of evidence recommendations**

**Supplementary appendix 3 A detailed process of development of minimized PR program**

**Supplementary appendix 4 Final draft of minimized PR program for older adults in long-term care facilities**

**Supplementary appendix 5 Components of the intervention**

**eFigure 1 Flow chart of PR implementation**

**Supplementary appendix 6 Qualitative themes of study**

**Supplementary appendix 1 A detailed process of the evidence summary**

We searched guidelines, best practice, evidence summary, recommended practice, expert consensus, and systematic review from relevant guideline websites. The date was from January 1, 2000, to July 15, 2020. This part titled *“Evidence summary for prevention and management of physical restraints among older adults in the long-term care facilities”,* is a part of the first author's dissertation (Wang, 2021)*.* The overview process of the evidence summary was as follows:

**(1) Databases searched in the study**

| **International Organisations** | **Links** |
| --- | --- |
| Uptodate | https://www.uptodate.com |
| BMJ Best Practice | https://bestpractice.bmj.com |
| G-I-N - Guidelines International Network | http://www.g-i-n.net |
| NICE - National Institute for Health and Clinical Excellence | http://www.nice.org.uk |
| SIGN - Scottish Intercollegiate Guidelines Network | http://www.sign.ac.uk |
| RNAO - Registered Nurses' Association of Ontario | https://rnao.ca/ |
| ANA - American Nurses Association | http://www.nursingworld.org |
| RCN - Royal College of Nursing | http://www.rcn.org.uk |
| ICN - International Council of Nurses | http://www.icn.ch |
| AGS - The American Geriatrics Society | http://www.americangeriatrics.org |
| Pubmed | https://pubmed.ncbi.nlm.nih.gov/ |
| CDSR - The Cochrane Database of Systematic Reviews | https://www.cochranelibrary.com |
| JBI - The Joanna Briggs Institute | http://www.joannabriggs.edu.au |
| **Chinese Organisations** | **Links** |
| Medlive | https://www.medlive.cn |
| CNA - Chinese Nursing Association | http://www.cna-cast.org.cn |
| CNKI - China National Knowledge Infrastructure | https://www.cnki.net |
| Wanfang Data | https://www.wanfangdata.com.cn |

**(2) Search strategies**

Based on existing literature in the field, the following keywords and Mesh terms were used to guide the search: (a) Mesh terms, “restraint, physical” “nursing home” “older adults” “guideline” “meta analysis”; (b) Keywords, “long-term care/nursing home*/care home/residential home*/residential fa-cilit*/long-term care setting*/skilled nursing facilit*” “physical restraint*/bedrail*/bedchair*/belt*/physical immobilization/mechanical restraint*/constraint*/restrict*” “aged/elderly/geriatric care” “recommendation/statement/guidance/handbook/manual/summary/principle” “systematic review/systematic review and meta analysis/meta-synthesis”

**(3) Selection of literature and data extraction**

All literature was reviewed for relevance by at least two independent researchers. Disagreements were resolved through a consensus. The PRISMA flow diagram was as follows.


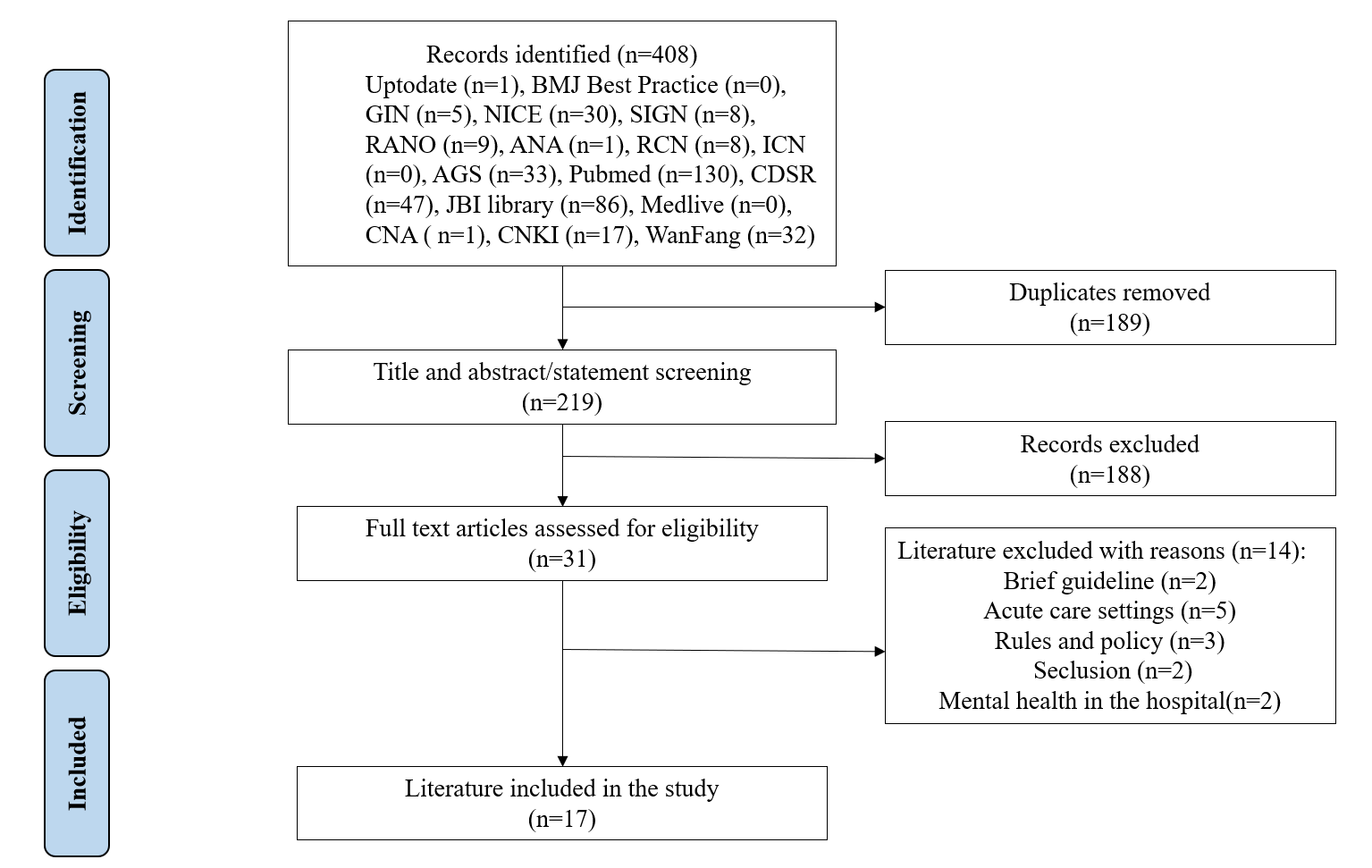


**(4) Appraising the quality of literature**

Included literature was independently evaluated by two researchers trained in evidence-based nursing, and evidence-based group discussions were performed in case of disagreement. In the event of conflicting conclusions from different sources of evidence, followed the principle of giving priority to evidence-based literature, authoritative literature, the latest published evidence, and high-quality evidence. Appraisal of Guidelines for Research and Evaluation (AGREEⅡ) was performed to appraise the guideline (Brouwers et al., 2010). A MeaSurement Tool to Assess systematic Reviews (AMSTAR 2) for appraising systematic review (Zhang et al., 2018). The JBI evidence-based health care center's experts' opinion and professional consensus evaluation tool was used to evaluate the authenticity and quality of expert consensus (Hu and Hao, 2019). The quality level of evidence and grade of recommendation was assessed by the JBI evidence pre-classification and evidence rank system (2014 Edition) (The Joanna Briggs Institute, 2014).

**(5) 17 pieces of literature included in the study**

1. Registered Nurses' Association of Ontario (RNAO). Promoting Safety: Alternative Approaches to the Use of Restraints. 2021; 2012. Available at: https://rnao.ca/bpg/guidelines/promoting-safety-alternativeapproaches-use-restraints.
2. Tania Marin MPH (BHSc). Evidence Summary. Physical Restraint Residential Aged Care (Effectiveness and Use). The Joanna Briggs Institute EBP Database, JBI@Ovid.JBI1115
3. Madhava Sai Sivapuram MS. Evidence Summary. Dementia (Older People): Reduction of Restrictive Practices (Workforce Competency Development). The Joanna Briggs Institute EBP Database, JBI@Ovid.JBI18605
4. Joanna Briggs Institute (JBI). Best Practice. Physical Restraint - Pt 1: Use in Acute and Residential Care Facilities. 2002;6(3):1-6
5. Joanna Briggs Institute (JBI). Best Practice. Physical Restraint - Pt 2: Minimisation in Acute and Residential Care Facilities. 2002;6(4):1-6
6. Joanna Briggs Institute (JBI). Recommended Practice. Restraint Standards. The Joanna Briggs Institute EBP Database, JBI@Ovid. 2017:JBI2153
7. Bellenger EN, Ibrahim JE, Kennedy B, Bugeja L. Prevention of physical restraint use among nursing home residents in Australia: The top three recommendations from experts and stakeholders. Int J Older People Nurs. 2019;14(1):e12218.doi:10.1111/opn.12218
8. Bleijlevens MH, Wagner LM, Capezuti E, Hamers JP. Physical Restraints: Consensus of a Research Definition Using a Modified Delphi Technique. J Am Geriatr Soc. 2016;64(11):2307-2310.doi:10.1111/jgs.14435
9. Australian and New Zealand Society for Geriatric Medicine Position Statement No 2 Physical Restraint Use in Older People. 2021; https://onlinelibrary.wiley.com/action/downloadSupplement?doi=10.1111%2Fajag.12224&file=ajag12224-sup-0001-Article_S1.pdf. Accessed 2016-05-25.
10. Mohler R, Richter T, Kopke S, Meyer G. Interventions for preventing and reducing the use of physical restraints in long-term geriatric care - a Cochrane review. J Clin Nurs. 2012;21(21-22):3070-3081.doi:10.1111/j.1365-2702.2012.04153.x
11. Hofmann H, Hahn S. Characteristics of nursing home residents and physical restraint: a systematic literature review. J Clin Nurs. 2014;23(21-22):3012-3024.doi:10.1111/jocn.12384
12. Kong EH, Choi H, Evans LK. Staff perceptions of barriers to physical restraint-reduction in long-term care: a meta-synthesis. J Clin Nurs. 2017;26(1-2):49-60.doi:10.1111/jocn.13418
13. Huynh D, Lee ON, An PM, Ens TA, Mannion CA. Bedrails and Falls in Nursing Homes: A Systematic Review. Clinical nursing research. 2020:1054773820907805.doi:10.1177/1054773820907805
14. Bellenger EN, Ibrahim JE, Lovell JJ, Bugeja L. The Nature and Extent of Physical Restraint–Related Deaths in Nursing Homes: A Systematic Review. J Aging Health. 2018;30(7):1042-1061.doi:10.1177/0898264317704541
15. Evans D, Wood J, Lambert L. A review of physical restraint minimization in the acute and residential care settings. J Adv Nurs. 2002;40(6):616-625.doi:10.1046/j.1365-2648.2002.02422.x
16. Lan SH, Lu LC, Lan SJ, et al. Educational intervention on physical restraint use in long-term care facilities – Systematic review and meta-analysis. Kaohsiung J Med Sci. 2017;33(8):411-421.doi:10.1016/j.kjms.2017.05.012
17. Tang Wing S, Chow Yeow L, Koh Siew Lin S. The effectiveness of physical restraints in reducing falls among adults in acute care hospitals and nursing homes: a systematic review. JBI Library of Systematic Reviews. 2012;10(5):307-351.doi:10.11124/jbisrir-2012-4

**Supplementary appendix 2 Overview of evidence recommendations**

| Dimension | Content | Level of evidence | Grade recommendation |
| --- | --- | --- | --- |
| Definition | 1. Consensus of a research definition for PR | Level 5 | A |
| Personnel requirement | 2. Trained and experienced professional staff to practice PR | Level5 | A |
|  | 3. Multidisciplinary teams to participate in PR management | Level3 | B |
| Evaluation | 4. Comprehensive assessment of restraint-related risk factors | Level3 | B |
|  | 5. Dynamic assessment in the whole process | Level4 | A |
|  | 6. With the help of comprehensive geriatric assessment tools and clinical experience | Level1 | A |
| Decision-making | 7. Consultative decision-making and authorization | level2 | B |
| Inform consent | 8. Obtaining informed consent | Level4 | A |
| Prevention strategies | 9. Implementing multi-component interventions to prevent and reduce PR | level1 | A |
|  | 10. Alternatives: physiological needs satisfaction, psychosocial care, improvement in management, environmental modification, electronic monitor and alarm systems, etc. | Level2 | B |
| Implementation | 11. PR may be used in emergencies or no alternative is available | Level5 | A |
|  | 12. Choose appropriate devices and use them correctly | Level4 | A |
| Monitoring | 13. Regular monitoring | Level5 | A |
|  | 14. Monitoring content includes restraints situations, adverse reactions, etc | Level4 | A |
| Removing | 15. Assessment of the continuing need for restraint and earliest possible removing | Level5 | A |
| Recording | 16. Documenting for record and inspection purposes | Level4 | B |
| Reporting | 17. Adopting a unified and systematic approach to investigate, analyze, and report PR | Level5 | B |
| Education | 18. Nursing curricula and continuous education | Level1 | A |
|  | 19. Applying multiple training methods | Level1 | A |
|  | 20. All staff involved in PR need to be trained, even older adults and families | Level1 | A |
|  | 21. Training content includes risk factors, implementation, alternatives, ethics, etc. | Level1 | A |
| Organization Policy | 22. Developing norms, procedures, and strategies for PR | Level4 | B |
|  | 23. Clarifying the definition and principles of PR | Level5 | B |
|  | 24. Resources support | Level5 | B |
|  | 25. Minimized restraint culture and goals | Level4 | B |

Notes: Physical restraint, PR

**Supplementary appendix 3 A detailed process of development of minimized PR program**

The first draft of this study was formed by literature analysis, group discussion, and evidence. The main comments of the group discussion were as follows:

(1) Adjusted practice-level content into evaluation, decision-making, informed consent, alternatives, implementation, monitoring, removing, recording, and reporting. Integrated the definition of restraint and the personnel requirement into the organizational support dimension.

(2) Refined the restraint indications and adjusted them to the evaluation dimension. The implementation dimension was refined into restraint tools selection, restraint tightness, restraint fixation, etc.

A total of 10 experts attended the meeting, including 3 males and 7 females, aged 36-57 years, with an average age of (45±4.67) years. The information of experts was as follows:

| **Number** | **Sex** | **Age** | **Education level** | **Job title** | **Years of work** | **Areas of expertise** |
| --- | --- | --- | --- | --- | --- | --- |
| 1 | Female | 57 | Undergraduate | Professor | 38 | Geriatric Nursing |
| 2 | Male | 46 | Undergraduate | Associate chief physician | 24 | Rehabilitation medicine |
| 3 | Female | 45 | PhD | Associate professor | 18 | Physical restraint |
| 4 | Female | 54 | Undergraduate | Associate professor | 34 | Quality control |
| 5 | Female | 39 | Master's degree | Nurse-in-charge | 15 | Geriatric Nursing |
| 6 | Male | 55 | Undergraduate | Associate chief physician | 32 | Geriatric Medicine |
| 7 | Female | 52 | Master's degree | Associate professor of nursing | 32 | Physical restraints |
| 8 | Female | 36 | PhD | Associate professor | 15 | Geriatric Nursing |
| 9 | Male | 56 | Master's degree | Chief physicians | 30 | Nursing management |
| 10 | Female | 38 | Undergraduate | Nurse-in-charge | 14 | Geriatric Nursing |

Expert opinions of meetings were listed below:

| **Expert opinions adopted** | **Numbers** |
| --- | --- |
| 1. Eliminating temporary medical orders of PR from physicians because of their poor operability | 4 |
| 2. Clarifying the time (i.e., admission or change in condition) on risk notification and informed consent | 2 |
| 3. A comprehensive assessment every 3 to 6 months and an updated informed consent form. | 1 |
| 4. Adding “staff training” at the improvement in management in the alternatives dimension. | 1 |
| 5. The frequency of monitoring should be based on the individual situation. Adding on-demand monitoring | 3 |
| 6. Adding specific indications of PR removal | 2 |
| 7. Developing a record sheet for documentation of PR | 3 |
| 8. Adding to informal training in practice, such as case discussions or analysis during ward rounds and handovers. | 1 |
| 9. If available, training should include older adults and their family members | 2 |
| 10. Developing a flow chart of PR implementation | 1 |
| 11. Adding overall principles of PR | 1 |
| 12. Clarifying the scope of responsibilities of nurses, and nursing assistants in the PR practice. | 1 |
| **Expert opinions not adopted** | **Numbers** |
| 1. The content of the assessment is very large and the workload is relatively large, which may be difficult to implement in actual operation  **Reasons:** The evaluation content is consistent with the comprehensive evaluation content in long-term care facilities, and it is practical and operable | 1 |
| 2. The content of the assessment is not an indication of PR, and scientific tools should be used to determine the need for PR  **Reasons:** Assessment content to identify the risk of PR and developing interventions; No assessment tool to identify whether to use or not in long-term care facilities | 2 |
| 3. Electronic monitor and alarm systems as alternatives with the high cost are low utilization  **Reasons:** Some smart facilities are available to use | 1 |

**Supplementary appendix 4 Final draft of minimized PR program for older adults in long-term care facilities**

| Dimension | Details |
| --- | --- |
| Restraint principle | 1. The principle of restraint-free or minimized physical restraint, that is, physical restraint is used only as an intervention of last resort when alternative solutions have failed.  2. Respect the dignity, privacy, culture, and personal rights of older adults. |
| Organizational support | 3. Define the concept: clarify the definition of physical restraint, such as the use of bedside rails to prevent older adults from getting out of bed or trying to leave the bed as a restraint, therapeutic devices to keep old adults alive are not included.  4. Set up a physical restraint management group including doctors, nurses, nursing assistants, rehabilitation therapist, dietitians, older adults, and their family members.  5. Formulate the implementation process of physical restraint for older adults in long-term care facilities  6. Incorporate physical restraints into indicators of institutional care quality, and regular spot checks, supervision, and quality improvement are recommended.  7. Provide resources, organization, and administrative support to implement programs to minimize physical restraint |
| Personnel requirement | 8. Assessment of physical restraint should be performed by a multidisciplinary team of trained nurses, physicians, and therapists.  9. Physical restraint should be practiced by experienced nurses or nursing assistants who have received specific training and dynamically monitored and recorded  10. The decision-making of physical restraint should be discussed with the physical restraint management team in the organization based on the comprehensive assessment results. |
| Evaluation | 11. The risk factors：①Individual factors include advanced age, cognitive impairment, behavioral reactions (aggression, violence, self-injury, agitation, wandering, etc.), fecal and urinary incontinence, the decline in the ability of daily living, mobility impairment, fall history or fall, fear, etc；②Organizational factors include institutional culture, available human resources, etc. ③Inducements include environmental factors, unmet demand, drug influence, etc. |
|  | 12. Assessment tools: Assess the specific physical restraint risk factors based on clinical experience and effective tools, such as the CMAI Agitated Behavior Scale, BVC violence assessment form, assessment tools of pain, cognitive function, and fall risk. |
|  | 13. Restraint indications：①Indication: hurting themselves and/or others in an emergency. Risk of falling, tube drawing, self-injury and injury to others when alternative measures are ineffective. Seriously interferes with life treatment in older adults when alternative measures are ineffective. ②Contraindications: Use of physical restraint for lack of personnel, seeking convenience, or punishing older adults. Older adults are conscious and cooperative; Body muscle strength ≤ level 2. Older adults in a coma. |
| Decision-making | 14. Identify high-risk older adults, and make decisions with the physical restraint management team, older adults, and their families. |
| Inform consent | 15. Sign the risk notification and informed consent when comprehensive evaluation at admission or when the condition changes.  16. Risk notification and informed consent should be re-signed after a comprehensive evaluation every 3 to 6 months. |
| Alternatives | 17. Alternatives:  ①Physiological aspects: needs satisfaction, improving sleep quality, relieving pain, etc.  ②Psychosocial care: increased companionship with peers, family, and volunteers; divert attention; relieve stress; communication and talking out appropriately encouraged; increase social interaction, etc.  ③Improvement in management: be familiar with the behavior of older adults and monitoring timely; Increase the frequency of inspections; rational allocation of nursing staff and improve their nursing ability, reduce workload; observe and assist activities of older adults timely; Special care for high-risk older adults  ④Environmental modification: provide a comfortable, safe, quiet environment; ensure safety devices such as beds and wheelchairs for older adults, etc.  ⑤Alternative devices: hip protectors, non-slip socks, foot or seat sensors, installed bedside alarms, or electronic monitoring systems, if available |
| Implementation | 18. Restraint tools：①In case of agitation or violence, restraint belts should be used to restrain limbs, strong, non-slip, and small shrinkage materials should be selected for restraint, and gauze should not be used for restraint. Soft restraint belts are suitable for limb restraint of those who have partial cooperation intention.  19. Restraint position: Limb restraints are commonly used. It is necessary to ensure full chest expansion and ventilation when chest restraints, avoid prone tying, and forbid head and neck restraining.  20. Degree of tightness: Skin protection and maintenance of functional position should be given to the restricted part, and the tightness of the restraint should be appropriate (1-2 fingers should be allowed).  21. Fixed methods: The restraint tool should be applied to the restraint part correctly and fixed properly. It can not be fixed on movable objects, such as the bedside rails. Ensure that the wheelchair or seat is securely secured when restrained. |
| Monitoring | 22. Monitoring frequency: The monitoring frequency should be determined according to the actual situation of older adults. In principle, the responsible nurse should check the physical restraint every 30~60min, release the limb restraint at least once every hour, and check the adverse reactions of the physical restraint every 3~4h. |
|  | 23. Monitoring indicators：①Conditions of physical restraint: tightness of restraint, the position of restraint, range of motion, etc；②Adverse reactions: Skin(color, temperature, integrity, sensory, circulation, etc), physiological requirements(hunger, thirst, cleanliness, comfort, posture, excretion, etc), emotion and behavior(fear, depression, agitation, delirium, etc), accidents (falls, tube drawing, etc). |
| Removing | 24. The necessity of physical restraint should be assessed daily and removed as soon as possible. Indications of removing: ①Improved consciousness, behavior; and communicative and cooperative; ②With level 3 and above muscle strength, coma or sedation；③Life-supporting treatment of older adults completed. |
| Recording | 25. Develop a physical restraint care record, including risk factors, implementation of alternative measures, restraint reason, restraint types, restraint position, start time, release time, and restraint complications. |
| Reporting | 26. Recommend a unified system approach to improve the identification, investigation, analysis, and report of PR-related adverse events. |
| Personnel training | 27. The training object: all staff in the institution, older adults, and their families. |
|  | 28. Training form：①Formal training, such as theoretical lectures, workshops, seminars, case studies, scenario simulations, video learning, etc.；②Informal training, such as ward rounds, physical restraint knowledge, and skills learning during shift exchange. |
|  | 29. Training content: all evidenced-updated information and clinical practice involving physical restraint, including risk factors, alternatives, ethics and law, nursing practice, special care for dementia, wandering, and fall-risk older adults |

Notes: CMAI, the Cohen Mansfield agitation inventory; BVC, Broset Violence Checklist

**Supplementary** **appendix 5 Components of the intervention**

| **Components** | **Description** |
| --- | --- |
| 1. Organizational support | 1.1 Unify rules and regulations: principles and definitions of physical restraint, nursing practice based on minimized physical restraint program.  1.2 Formulate the implementation process of the physical restraint **(eFigure 1)**  1.3 Develop a restraint management group (one doctor, two nurses, two nursing assistants, and one rehabilitation therapist) for determining the necessity of restraint use based on the content of the evaluation dimension in the minimized physical restraint program.  1.4 Declaration confirming that physical restraint is an indicator of institutional care quality, and conducts quality improvement discussion for regular spot checks, and supervision once a month.  1.5 Provide resources: qualified restraint devices, printed supportive training manual, regular group discussion. |
| 2. Education support | 2.1 60-minute program information and group discussion for leaders (i.e., one dean of the nursing home and one nursing manager of the geriatric ward), four nurses, and one rehabilitation therapist. The aim is to get their support and recommendations for program implementation.  2.2 Education for nursing assistants: four training sessions including three theoretical lectures and one operation training. Details were presented in Table 1 in the manuscript. |
| 3. Consultation | 3.1 Reinforce the principle of least restraint during communication, relieve the stress of individuals with fear of responsibility through co-decision and implementation guidance, etc., to support the nursing assistants in achieving restraint-free care in daily practice.  3.2 Discuss with the restraint management team to identify older adults at high risk for physical restraint and help in creating an individualized plan of care that focuses on alternative approaches.  3.3 Visit the nursing home once a week and provide two-hour ward rounds for informal training.  3.4 Attend quality improvement project meetings about reducing physical restraint per month.  3.5 Resolve issues related to institutional physical restraints at any moment, the problems that nursing assistants asked for help with were described in the manuscript. |

**eFigure 1 Flow chart of PR implementation**


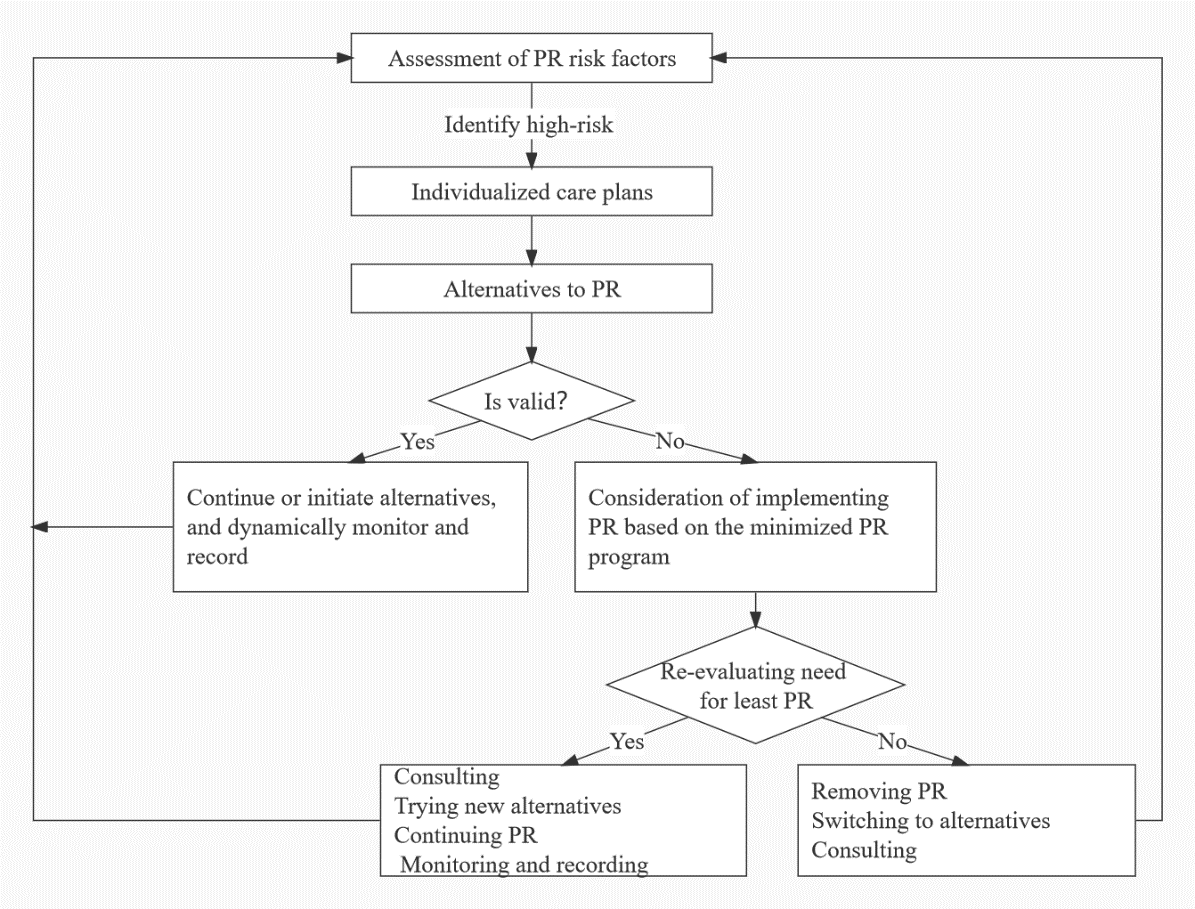


**Notes:** PR, physical restraint

To identify on admission and throughout an episode of care those older adults at risk for behaviors that may lead to harm to self/others, and seriously interfere with life treatment, that is, older adults at risk for using PR. A combination of clinical judgment with further evaluation based on the content of the evaluation dimension in the minimized PR program guides the identification of high-risk.

After we identified older adults with high risk, an individualized plan of care that focuses on alternative approaches to the use of restraints was created by the restraint management group. The suggested specific alternatives for specific risk behavior were based on alternatives recommended in the guideline titled “Promoting Safety: Alternative Approaches to the Use of Restraints” and best practice titled “Physical Restraint - Part 2: Minimisation in Acute and Residential Care Facilities”. Additionally, these alternatives were trained for nursing assistants in education plans.

The effectiveness of alternatives was estimated based on clinical judgment and review criteria for the least restraint principles. For example, alternatives could prevent older adults from serious harm to self/others and enhance older adults’ dignity, privacy, personal rights, and quality of life.

Re-evaluating the need for PR was performed based on the content of the evaluation, monitoring, and removing dimensions of the program. After re-evaluation, new risk factors may arise due to changes in the conditions of older adults, thus, new alternatives for identified risk behavior could be chosen for the least restraint.

**Supplementary** **appendix 6 Interview guide and qualitative themes of study**

**(1) Interview guide used in the qualitative descriptive study**

1. How would you evaluate the minimized physical restraint program?
2. What is your experience in the process of program implementation?
3. What are the barriers or challenges to the implementation of the program?
4. How do you think the program could be best applied in the nursing home?
5. Is there anything else you would like to add?

Notes: Questions may be supplemented with open-ended questions such as, “Could you describe more details about that?” or “Could you please give an example?”

**(2) Qualitative themes of study**

| **Themes** | **Sub-themes** | **Quotations** |
| --- | --- | --- |
| Learning and applying for the program | Knowledge update | 1. *“Learned about the types of restraint， how to implement, nursing practice, and its adverse effects, which were previously unknown” (NA-03)* 2. *“We didn't know there were so many principles of restraint, and we thought that the use depended on our experience”* *(NA-04)* 3. *“Physical restraint training has brought us a lot of new knowledge and deepened our understanding of* *restraint, for example, I previously thought restraint was a protective measure” (NA-07)* |
|  | Attitudes changes | 1. *“When I restrain them now, I will think of respecting older adults and paying attention to their emotions and needs at all times” (NA-01)* 2. *“I used to think restraint is for the safety of older adults and is a common practice, but this training has changed my opinion” (NA-07)* 3. *“This practice gradually changed our attitude towards the use of* *restraint ” (NA-12)* |
|  | Practice improvement | 1. *“Mastered some common risk management skills of older adults, such as falling and getting lost” (NA-09)* 2. *“learned the specific implementation methods of physical restraint, such as the choice of restraint, position placement, fixation” (NA-011)* 3. *“Realized that I had made some mistakes in restraining  lower limbs after the training, and I have corrected them in time” (NA-13)* |
| Experiencing two-side feelings | Confidence in the program | 1. *“This program gave us a lot of guidance on how to use restraints and gave me some confidence in how to use them properly” (NA-02)* 2. *“I felt that the physical restraints within the institution are becoming more standardized obviously, which is a great progress for the quality of care” (NA-08)* 3. *“Nursing staff have a stronger thirst for knowledge of restraint knowledge, they often ask and discuss with nurses when the older adults in their care need restraint” (Dean of the nursing home)* |
|  | Burden increases | 1. *“Sometimes there is a fear of accidents that don't restrain the elderly” (NA-01)* 2. *“I need to spend more time with older adults who have been released from restraint to care for them all the time” (NA-05)* 3. *“Now we have to fill out a physical* *restraint nursing record, which is a burden for us and doesn't feel very* *useful” (NA-06)* |
| Encountering challenges and barriers | Difficulty to assess the necessity of restraint | 1. “*Lack of the assessment tools effective and measurable, and the status of the elderly is constantly changing*” 2. *“It is difficult for the restraint group to assess comprehensively whether to use restraint whenever and wherever possible, and the actual workload is a little heavy” (Nursing manager of the nursing home)* |
|  | Poor feasibility of alternatives | 1. *“Alternative solutions are difficult to implement in practice and the effect is uncertain.” (NA-09)* 2. *“It is hoped that the alternatives of restraint can be more detailed and specific, such as which types of alternatives for older adults use and how are they implemented.” (NA-13)* |

**References**

Brouwers, M.C., Kho, M.E., Browman, G.P., Burgers, J.S., Cluzeau, F., Feder, G., et al. (2010). AGREE II: advancing guideline development, reporting and evaluation in health care. *Cmaj* 182(18)**,** E839-842. doi: 10.1503/cmaj.090449.

Hu, Y., and Hao, Y. (2019). "Evidence-based Nursing (2nd Edition)." Beijing: People's Medical Publishing House, 77.

The Joanna Briggs Institute (2014). *The Joanna Briggs Institute Levels of Evidence and Grades of Recommendation Working Party.Supporting Document for the Joanna Briggs Institute levels of Evidence and Grades of Recommendation* [Online]. The Joanna Briggs Institute. Available: http://joannabriggs.org/jbi-approach.html#tabbed-nav=Levels-of-Evidence [Accessed 2014].

Wang, J. (2021). *Evidence-based practice of minimized physical restraint pro-gram for older adults in medical-nursing facilities.* Master, Chongqing Medical University.

Zhang, F., Shen, A., Zeng, X., Qiang, W., and Jin, Y. (2018). An Introduction to AMSTAR 2: a critical appraisal tool for systematic reviews. *Chin J Evid Based Cardiovasc Med* 10(01)**,** 14-18.
